# Retraction Note: MiR-29b/Sp1/FUT4 axis modulates the malignancy of leukemia stem cells by regulating fucosylation via Wnt/β-catenin pathway in acute myeloid leukemia

**DOI:** 10.1186/s13046-026-03662-1

**Published:** 2026-02-13

**Authors:** Bing Liu, Hongye Ma, Qianqian Liu, Yang Xiao, Shimeng Pan, Huimin Zhou, Li Jia

**Affiliations:** 1 College of Laboratory Medicine, Dalian Medical University, 9 Lushunnan Road, Xiduan, Dalian, Liaoning Province 116044 China; 2 Department of Clinical Laboratory, Beijing Hospital of Traditional Chinese Medicine Affiliated to Capital University of Medicine Sciences, Beijing, 100010 China; 3 Department of Microbiology, Dalian Medical University, Dalian, Liaoning Province 116044 China

**Retraction Note: J Exp Clin Cancer Res 38, 200 (2019)**

**https://doi.org/10.1186/s13046-019-1179-y**

The Editor-in-Chief has retracted this article. Concerns were raised regarding a number of figures, specifically:

- Figure 5d: the panel for CyclinD1 appears to overlap with the VVA-binding MUC1 panel in Fig. 6e of [1]; a correction was issued for the Fig. 5d CyclinD1 panel.
- Supplementary Fig. 1a: the panel for FUT4 appears to partially overlap with panel 2 in Fig. 2g of [2].

The Editor-in-Chief therefore no longer has confidence in the reliability of the data reported in the article.

Author Li Jia has stated on behalf of all authors that they disagree with this retraction.
